# PPARβ/δ Agonist GW501516 Inhibits Tumorigenicity of Undifferentiated Nasopharyngeal Carcinoma in C666-1 Cells by Promoting Apoptosis

**DOI:** 10.3389/fphar.2018.00648

**Published:** 2018-06-28

**Authors:** Yangyang Ji, Hui Li, Fang Wang, Linglan Gu

**Affiliations:** Department of ENT, Central Hospital of Minhang District (Minhang Hospital Fudan University), Shanghai, China

**Keywords:** nasopharyngeal carcinoma, PPARβ/δ, GW501516, apoptosis, AMPKα

## Abstract

Activation of peroxisome proliferator-activated receptor β/δ (PPARβ/δ) had been linked to inhibition on the proliferation and apoptosis in a few cancer cell lines. However, limited data exists regarding the role of PPARβ/δ in nasopharyngeal carcinoma (NPC). This study was undertaken to determine the effect of PPARβ/δ on cell proliferation, anchorage-dependent clonogenicity, and ectopic xenografts in the human NPC cell lines. Gene and protein expression of PPARβ/δ were reduced specifically in the poor- and un-differentiated NPC cell lines as compared with the control NP-69 cells. Ligand activation of PPARβ/δ by GW501516, a specific PPARβ/δ selective agonist, inhibited cell proliferation and colony formation strikingly, and induced a G2/M phase arrest in the EBV positive undifferentiated NPC C666-1 cells relative to the control cells. Moreover, GW501516 induced C666-1 cell apoptosis in a caspase and BAX dependent manner. In accordance with the *in vitro* result, GW501516 significantly suppressed the ectopic NPC xenograft tumorigenicity that derived from the C666-1 NPC cells in BALB/c nu/nu mice. This effect is greatly associated with its inhibition on the gene and protein expression of integrin-linked kinase (ILK) through activation of the AMPKα-dependent signaling pathways. Collectively, we showed that PPARβ/δ expression is in reverse correlation with the degree of differentiation in the NPC cell lines, and revealed the anti-tumorigenic effects of GW501516 in NPC cells by activation of AMPKα. This study suggested that PPARβ/δ targeting molecules may be useful for the poor-, and particularly un-differentiated NPC chemoprevention.

## Introduction

Nasopharyngeal carcinoma (NPC), a malignant carcinoma arising from the epithelial lining of the nasopharynx, is constantly associated with the Epstein Barr Virus (EBV), although only the EBV genome instead of viral particles was observed in the nucleus of the malignant NPC cells (Shannon-Lowe et al., 2009). As a multifactorial disease, NPC presents as a major public health problem throughout the world, particularly in East and Southeast Asia (Mahdavifar et al., 2016). Although NPC is relatively radiosensitive and chemosensitive among head and neck tumors, there are about 19–69% of patients will still experience the recurrence of NPC after initial treatment (Yang et al., 1996; Li et al., 2010; Suárez et al., 2010; Kong and Lu, 2016). However, effective treatments for them are still destitute in clinic. Hence diversified therapeutic agents for systemic treatments or as adjuvant chemotherapies to amplify currently available treatment protocols are thus desperately needed for improving the management of NPC (Kong and Lu, 2016). Novel approaches targeting of pivotal gene products whose function directly drives NPC will facilitate this process. However, molecular targeted therapy is still unavailable in the therapy of NPC, and substitute treatment options also remain rather poor (Zhang et al., 2013; Tan et al., 2016).

PPARβ/δ is one isotope of the nuclear hormone receptor peroxisome proliferator-activated receptors (PPARs), exerts pleiotropic biological functions ranging from regulation of lipid and glucose catabolism to modulation of cell proliferation and differentiation (Bedu et al., 2005; Peters and Gonzalez, 2009; Wagner and Wagner, 2010). It is recognized as a promising molecular target for chemoprevention of certain types of cancers (Peters et al., 2015b). A large body of evidence revealed that ligand activation of PPARβ/δ can prevent tumor promoting inflammation and induce terminal differentiation of cancer cells (Aung et al., 2006; Yang et al., 2010; Peters et al., 2015b), which could reverse sustained cell proliferation and promote sensitivity to growth suppressors (Aung et al., 2006; Peters and Gonzalez, 2009). Selective PPARβ/δ agonist GW501516 had been demonstrated to be able to inhibit the proliferation and promote the apoptosis in human endometrial cancer cells, human colonocytes, T24 urothelial cancer cells and C20 mouse mammary gland cancer cells (Matthiessen et al., 2005; Péchery et al., 2016), although it also exerted anti-apoptotic effects in some cell lines (such as cardiac myocytes, pancreatic β cells, hepatic stellate cells and colon cancer cell lines) (Wang et al., 2004; Barlaka et al., 2015). Additionally, GW501516 also showed activities of promoting proliferation in some kind of cancer cells, such as human non-small cell lung carcinoma and colorectal cancer cells (Han et al., 2008). Nevertheless, the expression of PPARβ/δ in NPC cells was still unclear till now, and whether PPARβ/δ is associated with the proliferation or apoptosis of NPC cells have never been explored.

In current study, we explored the expression of PPARβ/δ in human NPC cell lines with different degree of differentiation, and investigated the impact of GW501516 on proliferation, clonogenicity, and ectopic xenograft in the human NPC cell lines and analyzed the underlying mechanisms involved. Herein we found PPARβ/δ expression is in reverse correlation with the degree of differentiation in the NPC cell lines, the most striking reduction was observed in the EBV positive undifferentiated NPC C666-1 cells, where GW501516 treatment could inhibit the growth of NPC cells at both *in vitro* and *in vivo* level, through impairing cell cycle progression and promoting apoptosis by activation of the AMPKα and downregulation the expression of integrin-linked kinase (ILK).

## Materials and Methods

### Compounds

PPARβ/δ selective agonist GW501516 and PPARβ/δ selective antagonist GSK3787 were purchased from MedChemExpress (NJ, United States). The AMPK inhibitor compound C was obtained from Sigma-Aldrich (St. Louis, MO, United States).

### Cell Cultures and Reagents

Epstein Barr Virus-negative HK1 and CNE1 cell lines were bought from Institute of Virology, Chinese Academy of Preventive medicine, CNE2 and NP-69 cells were from the Shanghai Institute of Cell Biology (Shanghai, China), and the EBV-positive (C666-1) NPC cell line was purchased from the cell bank of Xiangya Central Laboratory (Central South University, Changsha, China). Cells were maintained in RPMI-1640 or DMEM/F12 (1:1) medium (Gibco, Thermo Fisher Scientific, Inc., Waltham, MA, United States) containing 100 U/ml penicillin, 100 μg/ml streptomycin, and supplemented with 10% fetal bovine serum (Gibco, Thermo Fisher Scientific, Inc.). C666-1 cell culture medium was additionally supplemented with 25 mM HEPES. Cells were cultured at 37°C in a humidified incubator with 5% CO_2_.

### PPARβ/δ Overexpression in C666-1 Cells

C666-1 cells seeded in 6-well plates were infected by adenoviruses PPARβ/δ (Ad-PPARβ/δ, 6 × 10^10^ pfu/mL) containing rat PPARβ/δ cDNA or adenovirus with human green fluorescent protein (GFP) (Ad-GFP, 4 × 10^10^ pfu/mL) as a control to Ad-PPARβ/δ, when the cells reached 75% confluence for 48 h. These two kinds of recombinant adenoviruses were produced by Genechem (Shanghai, China). The infection efficiency was monitored via fluorescence microscopy by the means of expressed GFP. Cell viability was assayed by MTT method to determine the impact of PPARβ/δ overexpression on cell viability. The protein expression level of PPARβ/δ was detected by western blot.

### RNA Extraction and Quantitative Polymerase Chain Reaction (QPCR)

Total RNA from cells was extracted by Trizol reagent (Invitrogen, Carlsbad, CA, United States), and reversely transcripted to cDNA with High Capacity cDNA Reverse Transciption Kit (Applied Biosystems, Foster City, CA, United States) in accordance to the manufacturer’s instruction. Then QPCR was performed on an ABI 7500 Real-time PCR system (Applied Biosystems, Foster City, CA, United States) with the power SYBR Green PCR Master Mix (Applied Biosystems, Warrington, United Kingdom). The primers used for QPCR is shown in **Table 1**. The level of β-actin was used as an internal control, and the level of PPARβ/δ was presented as relative expression of transcripts normalized against β-actin. Fold changes in expression were calculated using the method of 2^-ΔΔ^*^C^*^t^.

**Table 1:** The primer sequences used for qPCR.

Genes	Forward primers	Reverse primers
PPARβ/δ	5′-TCCAGAAGAAGAACCGCAACA-3′	5′ -GGATAGCGTTGTGCGACATG-3′
ILK	5′ ATGAAGACCCTGCAAAGCGA-3′	5′ -GAGTTTGGGCAAGGACCTGA-3′
β-actin	5′-TAAAGACCTCTATGCCAACACAG-3′	5′ -CACGATGGAGGGGCCGGACT-3′


### Methylthiazolyl Blue Tetrazolium (MTT) Assay

Methylthiazolyl blue tetrazolium spectrophotometric dye assay was used to assess the impact of GW501516 to the cell viability. Briefly, cells were seeded into 96-well plate at a density of 3 × 10^4^ cells/well and treated with different concentrations of GW501516 or vehicle (0.5% DMSO containing medium) for 48 h. Then the cells were incubated for 3 h at 37°C with sterile MTT labeling dye (0.5 mg/mL, Sigma-Aldrich, St. Louis, MO, United States). After removal of the culture medium and addition of dimethyl sulfoxide, the absorbance was measured at 550 nm with a scanning multi-well spectrophotometer (ELISA reader, Perkin Elmer). All experiments were performed in triplicates.

### Colony Formation Assay

Cells were seeded in 6-well plated (500 cells/well) and treated with GW501516 for 15 days. Then the colonies were fixed with 4% formaldehyde for 5 min and stained with 0.5% crystal violet for 30 s. Visible cell colonies were counted and the results were shown as the fold change relative to that of vehicle control group.

### Flow Cytometry Analysis

Cell cycle and apoptosis assay were determined by flow cytometry as previously reported. Briefly, for the cell cycle analysis, the collected cells were fixed overnight at 4°C in cold 70% ethanol in phosphate buffer saline (PBS). After fixation, cells were washed with PBS, and stained with 3 μM propidium iodide (PI; Sigma-Aldrich; Merck KGaA) complemented with 50 μg/mL RNase at room temperature for 15 min. Then the DNA content was analyzed by flow cytometry using an excitation wavelength set at 488 nm and emission at 610 nm (FC 500, Beckman Coulter). The cell cycle was divided into G0/G1, S, and G2/M phases, based on the extent of DNA staining.

Cell apoptosis analysis was performed with the Alexa Fluor 488 Annexin V/Dead Cell Apoptosis Kit. C666-1 cells seeded at a density of 8 × 10^4^ cells/mL in 6-well plate were cultured overnight and then treated with GW501516 for 48 h. After harvesting via centrifugation, the cells were stained with Annexin V and PI according to the manufacturer’s instruction, and then measured by FACSCalibur Cytometer (BD Biosciences, CA, United States). According to the extent of staining by Annexin V or/and PI, cells were classified as “survival,” “early apoptosis,” “late apoptosis,” and “necrosis.”

### Cell Protein Extraction and Western Blot

Proteins from cultured cells were extracted with RIPA lysis buffer containing a protease inhibitor cocktail, and protein concentration was determined by BCA Protein Assay Kit (Pierce Biotechnology, IL, United States). Protein expression was measured by western blot. Briefly, equal amounts of protein (30 μg) were resolved by SDS-PAGE, and proteins were transferred onto polyvinylidene difluoride (PVDF) membranes. After blocking for 1 h at room temperature with either 5% non-fat milk or BSA in Tris-buffered saline, the membrane was incubated overnight at 4°C with the primary antibodies. The membranes were then washed with TBST (Tris-Buffered Saline Tween 20) and followed by horseradish peroxidase-conjugated secondary antibody (Santa Cruz Biotechnology, Santa Cruz, CA, United States). Signals were developed using Immobilon Western Chemiluminescent HRP Substrate (Millipore, Billerica, CA, United States) according to the instructions from the manufacturer. Antibodies against PARP 1 was purchased from Invitrogen (C-2-10; Calbiochem, San Diego, CA, United States). Microtubule associated protein 1 light chain 3β (LC3), total and phospho-AMPK (Thr172) were obtained from Sigma (St. Louis, MO, United States). Bcl-2, Bax, caspase-3, caspase-8, and caspase-9 antibodies were purchased from Cell Signaling Technology Inc. (Danvers, MA, United States). Antibodies against PPARβ/δ and GAPDH were purchased from Santa Cruz Biotechnology (Santa Cruz Biotechnology, Santa Cruz, CA, United States).

### Subcutaneous Xenograft Mice Model

8-week-old female BALB/c nu/nu mice (Shanghai Slike Experimental Animals Co., Shanghai, China) were maintained in pathogen-free conditions under controlled temperature and humidity with 12-h light/dark cycles, supplied with standard rodent food and water *ad libitum*. This study was carried out strictly in accordance with the recommendations of the Guide for the Care and Use of Laboratory Animals of the National Institutes of Health. The protocol for animal study was approved by the Institutional Animal Care and Use Committee of Fudan University. Approximately 1 × 10^7^ C666-1 cells were injected subcutaneously into the right axilla of mice. When the tumor grew to a volume of 30–50 mm^3^, the mice were randomly assigned into vehicle control (saline containing 0.5% DMSO) or GW501516 (10 mg/kg and 30 mg/kg) treatment groups (*n* = 8). Compound was given by intraperitoneal injection once per day for 4 weeks. Tumor volume during treatment was measured weekly with slide calipers, and volumes were calculated as length × width × width × 0.5. After all the experiments were completed, the mice were euthanized and tumor weights were measured.

### Statistical Analysis

Data were expressed as means ± SD. Statistical significance were assessed by Student’s *t*-test or one-way ANOVA followed by the Tukey’s multiple comparison tests with SPSS 19.0 software (SSPS Inc., Chicago, IL, United States). *P* < 0.05 and *P* < 0.01 were considered statistically significant.

## Results

### Expression of PPARβ/δ in NPC Cell Lines

**FIGURE 1:**
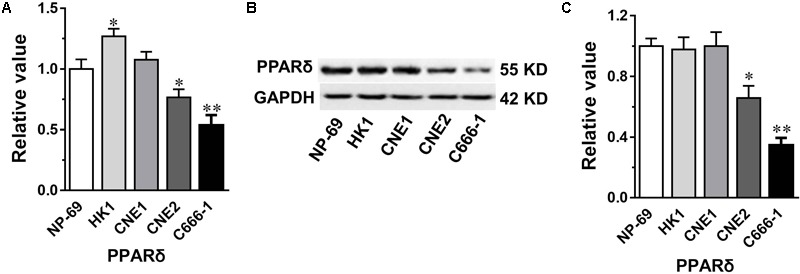
The gene and protein expression of PPARβ/δ in nasopharyngeal carcinoma (NPC) cell lines. **(A,B)** The gene and protein expression of PPARβ/δ in different NPC cell lines. Gene expression results are normalized to β-actin in the correspondent group, and then expressed as relative expression compared with that in the NP-69 group. **(C)** Densitometry analysis result of western blots in **(B)**. GAPDH was served as the loading control. Values are mean ± SD, *n* = 3. ^∗^*P* < 0.05, ^∗∗^*P* < 0.01 versus the NP-69 control group.

Based on the degree of differentiation, NPC is classified into well-, moderate- and un-differentiated carcinoma in clinic. Three type of NPC cell lines that could represent the three-tier histological classification of NPC were selected here to determine the gene and protein expression of PPARβ/δ under condition of NPC through QPCR and western blot analysis. Which include three EBV-negative (HK1, CNE1, CNE2) and one EBV positive (C666-1) cell lines. Interestingly, compared with the control NP-69 cells, strikingly reduced gene and protein expression of PPARβ/δ were only observed in the poor-differentiated CNE2 cells (*p* < 0.05 for both), and particularly in the undifferentiated EBV positive C666-1 cells (*p* < 0.01 for both) (**Figures 1A–C**). While no decrease was observed on expression of PPARβ/δ mRNA and protein in the two well differentiated EBV-negative (HK1, CNE1) NPC cell lines, and even a slight increase on PPARβ/δ mRNA expression was found in the HK1 cells (*p* < 0.05). Thus it can be concluded that PPARβ/δ expression was reduced in the poor- and un-differentiated NPC cell lines, and its expression seems in association with the degree of differentiation of the NPC.

### GW501516 or PPARβ/δ Overexpression Decreased Cell Growth in C666-1 Cells

**FIGURE 2:**
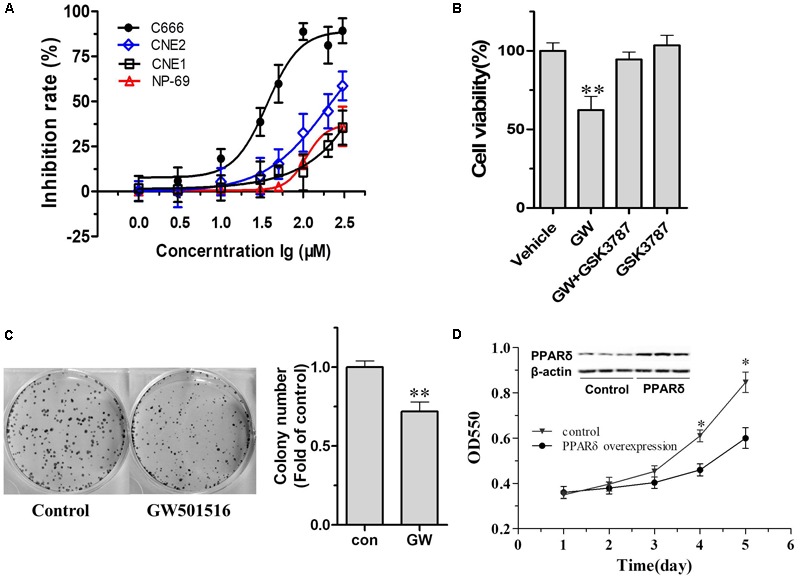
The impact of GW501516 on cell proliferation and colony formation. **(A)** The impact of GW501516 on cell proliferation in different NPC cell lines. The cell growth rates were determined by MTT after treatment with indicated concentration of GW501516 for 72 h. **(B)** PPARβ/δ antagonist GSK3787 antagonized GW501516 (GW) induced cytotoxic effect in C666-1 cells. 1 μM GSK3787 was co-administered with 30 μM GW501516 for 72 h, and then the cell viability was determined by MTT assay as indicated. **(C)** Representative image of colony formation assays of GW501516 treated C666-1 cells and the correspondent quantification result. **(D)** The impact of PPARβ/δ overexpression on cell proliferation in C666-1 cells. The inset was the representative image of PPARβ/δ at 48 h after transfection with Ad-PPARβ/δ or Ad-GFP (control) in C666-1 cells (*n* = 3 per group). Values are mean ± SD, *n* = 3. ^∗^*P* < 0.05, ^∗∗^*P* < 0.01 versus the control group.

We then examined the impact of PPARβ/δ activation on the growth of NPC cell lines with different degree of differentiation by treating the cells with a specific PPARβ/δ agonist. After 72-h treatment with various concentrations of GW501516. Dose-response curves in **Figure 2A** revealed GW501516 led to a distinct growth inhibition on the different NPC cell lines. Among them, the EBV-negative well-differentiated CNE1 was relatively resistant to GW501516, the maximum inhibition was around 40% observed at 300 μM, whereas no apparent impact was found at concentrations lower than 100 μM. However, this seems to be a non-specific cytotoxic effect induced by the higher concentration GW501516, because similar growth inhibition rate was also observed on the control NP-69 cells. Whereas the growth of the poor-differentiated CNE2 cells showed partially inhibition by GW501516, the difference on inhibition rate is around 20% compared with that of the NP-69 cells at concentrations from 30 to 300 μM (**Figure 2A**). In contrast, the undifferentiated EBV-positive C666-1 NPC cells was more sensitive, they showed slowest growth rate in the assay, a significant inhibition was found from 30 μM (38.58%) on, and the IC_50_ is calculated to be 36.31 μM. More importantly, the inhibiting effect induced by 30 μM GW501516 could be antagonized almost totally by combination with a specific PPARβ/δ antagonist GSK3787 (*p* < 0.05, vs. GW501516) (**Figure 2B**), indicating the growth inhibition effect of GW501516 is realized through activation of PPARβ/δ receptor. In corresponding with the above result, GW501516 also markedly reduced the colony numbers of C666-1 cells in the clonogenic assay (**Figure 2C**). Additionally, similar to the effect of PPARβ/δ activation, over-expression of PPARβ/δ also lead to a significant inhibition on the proliferation of C666-1 cells (**Figure 2D**). These results together signified that PPARβ/δ activation or over-expression could effectively attenuate the undifferentiated NPC cell proliferation and colony formation *in vitro*.

### GW501516 Impaired Cell Cycle Progression in C666-1 Cells

**FIGURE 3:**
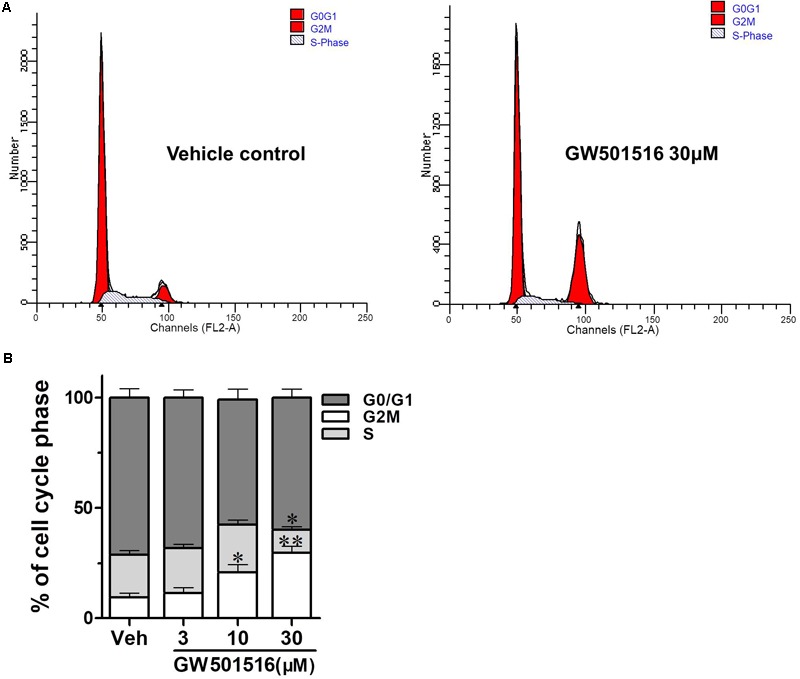
The impact of GW501516 on cell cycle of C666-1 cells. **(A)** Flow cytometry analysis showed that GW501516 impairs cell cycle progression in C666-1 cells. After treatment with indicated concentration of GW501516 for 72 h, the cell cycle was analyzed by the flow cytometry. **(B)** Calculated cell cycle distribution in C666-1 cells after treatment with indicated concentration of GW501516. Values are mean ± SD. ^∗^*P* < 0.05, ^∗∗^*P* < 0.01 versus the control group.

To further explore the underlying mechanisms of PPARβ/δ activation on inhibition of cell proliferation and colony formation, we then explored the impact of GW501516 on cell cycle distribution in C666-1 cells, the DNA content was measured by flow cytometry in C666-1 cells treated with 3, 10, and 30 μM GW501516 for 48 h. As showed in **Figure 3**, although lower dose (3 μM) of GW501516 did not modify the cell cycle distribution, the G2/M fraction of cells was significantly increased to 20.89 ± 3.43% and 29.62 ± 3.12%, respectively, in 10 and 30 μM GW501516 treatment group, from 9.53 ± 1.95% in the vehicle-treated control cells. Meanwhile, a slightly decreased cell distribution at S phase was observed in 30 μM GW501516 treated cells (19.25 ± 1.96% vs. 10.58 ± 1.24%, *p* < 0.05) (**Figure 3B**). These data indicated that the G2/M cell cycle arrest was associated with an increased cell death induced by higher dose of GW501516.

### GW501516 Induced Apoptosis of C666-1 Cells

**FIGURE 4:**
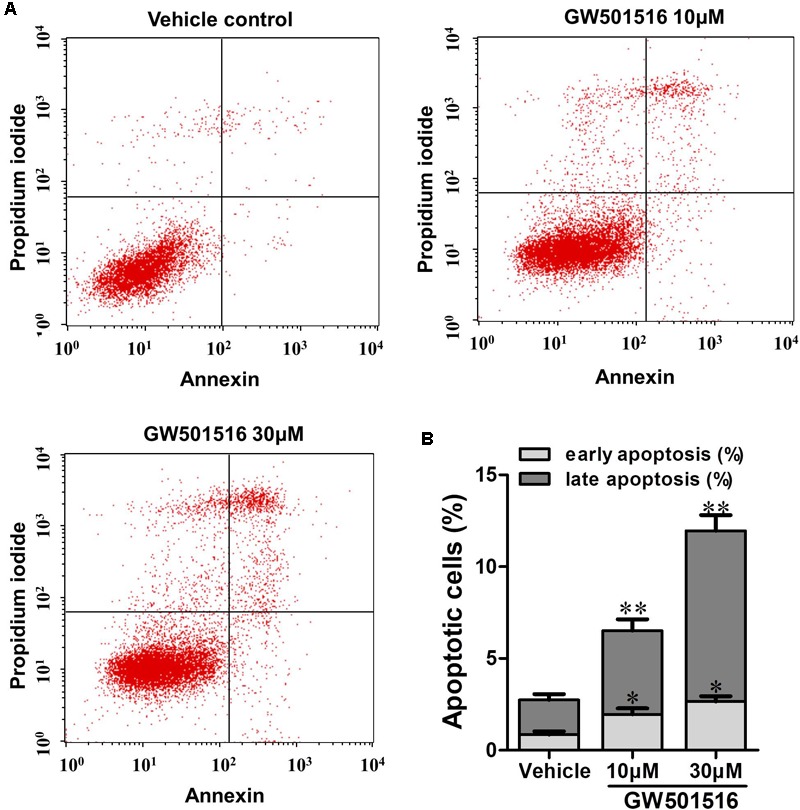
The impact of GW501516 on apoptosis in C666-1 cells. **(A)** Cells were treated with 10 or 30 μM GW501516 for 72 h and then assayed with flow cytometry. **(B)** The bar graph shows a significant increase in the apoptosis rate of GW501516 treated C666-1 cells. Values are mean ± SD. ^∗^*P* < 0.05, ^∗∗^*P* < 0.01 versus the control group.

To further investigate the impact of PPARβ/δ activation on C666-1 cell apoptosis, Annexin V-FITC/PI double staining was performed on GW501516 treated cells with the flow cytometry (FCM). As can be seen in **Figure 4**, after 48 h treatment, GW501516 induced C666-1 cell apoptosis in a concentration-dependent manner, the percentage of early and late apoptotic cells increased to 6.52 and 11.96% by 10 and 30 μM GW501516 (*p* < 0.01 for both), respectively. Which is significantly higher than that of the vehicle treated control cells (1.37%), suggesting apoptosis of C666-1 cells was promoted by GW501516.

### The Impact of GW501516 on the Expression of Apoptotic Associated Proteins in C666-1 Cells

**FIGURE 5:**
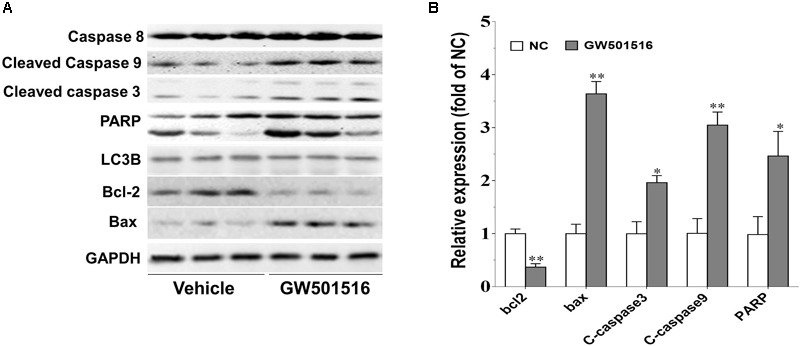
The impact of GW501516 on protein expression in C666-1 cells. **(A)** Western blot analysis of the protein expression of caspase and Bcl-2 family members in response to GW501516. After treatment with 30 μM GW501516 for 72 h, cells were harvested, lysed, and processed for western blot assay as described in Section “Materials and Methods.” **(B)** Quantification result of **(A)**. Values are mean ± SD. ^∗^*P* < 0.05, ^∗∗^*P* < 0.01 versus the control group.

A compound can induce apoptosis in tumor cells through several mechanisms, such as blocking the cell cycle (Yang et al., 1996), inhibiting the phosphorylation of Bcl-2 and Bcl-xl (Chen et al., 2015), activating caspases (Wagner and Wagner, 2010), up-regulating E2F1 (Huang et al., 1980), and causing the release of cytochrome c. We next detected the impact of GW501516 on the impact of caspases, since caspases are known to play critical roles in the initiation and maintenance of apoptosis. As shown in **Figure 5** the expression levels of cleaved caspase 3 and caspase 9 were both elevated after GW501516 treatment for 48 h in C666-1 cells (*p* < 0.05 and *p* < 0.01, respectively). In consistent with this, the downstream target of caspase 3, cleaved PARP was also increased exorbitantly (*p* < 0.05). In addition, the level of Bcl-2 was decreased markedly (*p* < 0.01), while the levels of Bax was significantly increased (*p* < 0.01). However, no impact on the expression of autophagy related protein LC3B was observed under such condition.

### GW501516 Inhibited Growth of NPC Xenograft Tumor in Nude Mice

**FIGURE 6:**
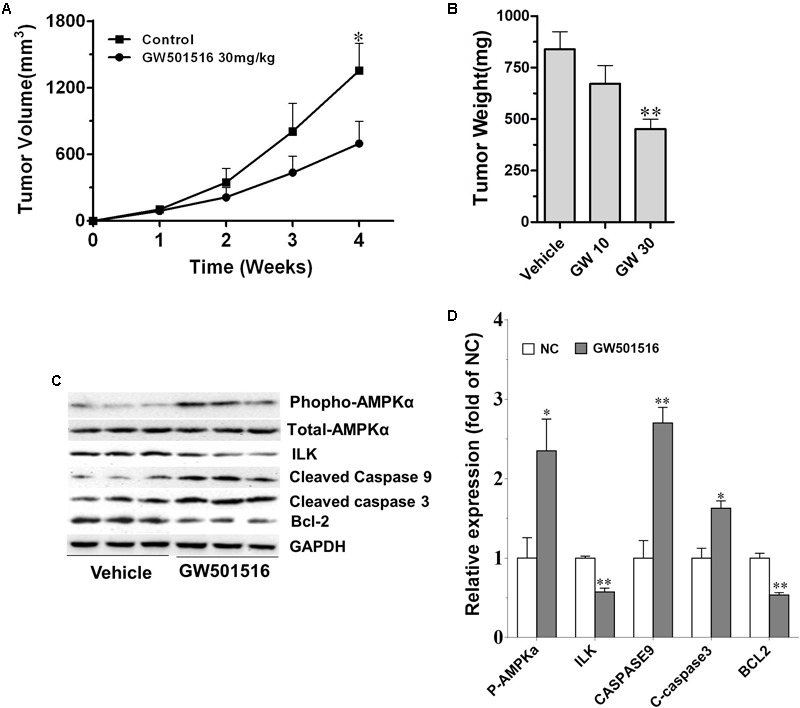
GW501516 suppressed SS tumor growth *in vivo*. **(A)** Tumor volume growth curve during vehicle or GW501516 treatment. Mice were treated with 10 or 30 mg/kg GW501516 for 4 weeks, and tumor volume during treatment was measured weekly. GW10, GW501516 10 mg/kg; GW30, GW501516 30 mg/kg. **(B)** Tumor weight measured at the end of the experiment. **(C,D)** Protein expression in C666-1 NPC xenograght samples and the corresponding quantification results. Values are mean ± SD. ^∗^*P* < 0.05, ^∗∗^*P* < 0.01 versus the control group.

The *in vivo* antitumor effect of GW501516 was further investigated using the mouse tumorigenicity assay. **Figures 6A,B** showed that GW501516 significantly decreased growth and weight of subcutaneous xenograft tumors when comparing with the vehicle treated control mice. The average tumor volume reached 200 mm^3^ at week 3, but did not exceed 600 mm^3^ until week 4, in contrast to that exceeded 1300 mm^3^ by week 4 in control mice (**Figure 6A**). To confirm the underlying mechanism by which GW501516 suppressed tumor formation in the BALB/c nu/nu mice, we analyzed the expression of apoptotic associated proteins in the C666-1 xenografts tumor samples that had proved to be modified by GW501516 at *in vitro* (**Figure 5**). In consistent with the *in vitro* result, cleaved caspase 3 and caspase 9 were increased significantly (*p* < 0.05 and *p* < 0.05, respectively), while Bcl-2 content was decreased strikingly (*p* < 0.01) (**Figures 6C,D**).

**FIGURE 7:**
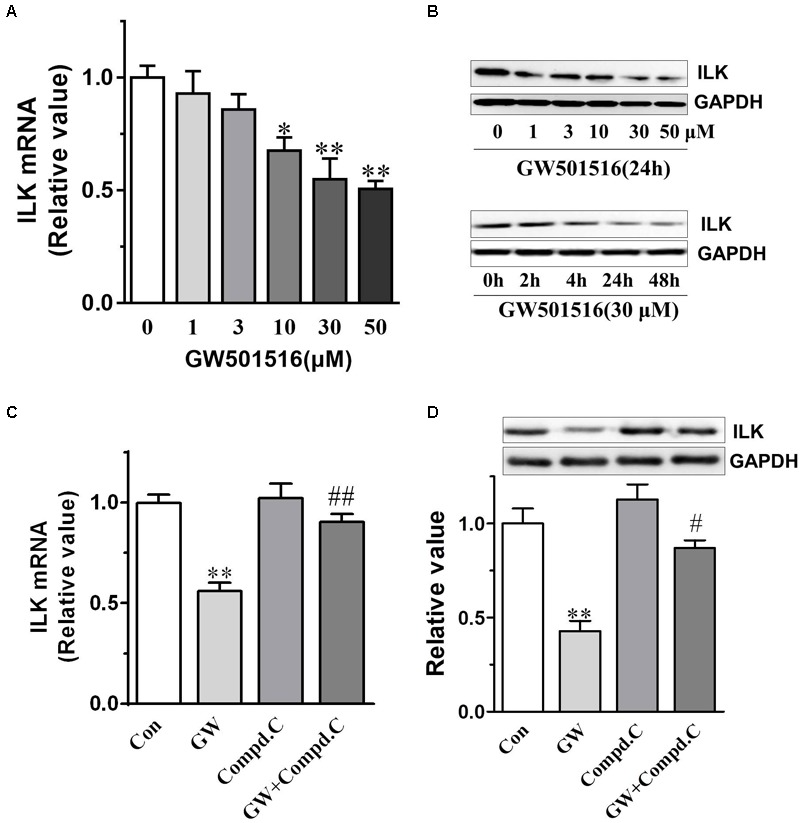
GW501516 inhibited gene and protein expression of integrin-linked kinase (ILK) in C666-1 cells. **(A)** ILK mRNA expression in C666-1 cells. Cells were exposed to indicate concentrations of GW501516 (GW) for 24 h and then gene expression of ILK were analyzed by QPCR. Gene expression results are normalized to β-actin in the correspondent treatment group, and then expressed as relative expression compared with that in the vehicle group. **(B)** ILK protein expression in C666-1 cells after treatment with increasing concentration of GW501516 for 24 h (upper panel), or treated with 30 μM GW501516 for the indicated time (lower panel). **(C,D)** AMPK inhibitor compound C (Compd.C) abrogated the suppressing effect of GW501516 on ILK gene and protein expression in C666-1 cells. Cells were treated with compound C (20 μM) for 30 min before exposure to GW501516 (30 μM) for an additional 24 h. After the incubation, RNA and protein were extracted from cells for QPCR and western blot assays, respectively. GAPDH was used as loading control in the western blot. *n* = 3, values are mean ± SD. ^∗^*P* < 0.05, ^∗∗^*P* < 0.01 versus the control (Con.) group. ^#^*P* < 0.05, ^##^*P* < 0.01 versus the GW group.

A recent study by Hahn et al. (2014) indicated that PPARγ agonist rosiglitazone inhibited NPC cell growth by reducing the expression of ILK through activation of PPARγ- and AMPKα. Meanwhile, previous studies had confirmed GW501516 could activate AMPKα-dependent signaling pathways in human skeletal myotubes and human keratinocytes (Krämer et al., 2005, 2007; Barroso et al., 2011). We thus examined whether GW501516 affect the phospho-AMPK and ILK levels in the C666-1 xenografts tumor samples (**Figures 6C,D**). Compared with the vehicle treated control, we found GW501516 treatment increased phospho-AMPK levels (*p* < 0.05) (**Figures 6C,D**). Meanwhile, ILK protein expression was markedly down-regulated by GW501516 (*p* < 0.05). To confirm the specificity of GW501516 on regulation the expression of ILK, we treated C666-1 cells with GW501516 at an *in vitro* level, and then analyzed the gene and protein expression of ILK with QPCR and western blot, respectively. **Figure 7A** showed that GW501516 down-regulated ILK mRNA expression dose-dependently. In line with this, ILK protein level in the C666-1 NPC cells was proved to lower in a dose- and time dependent manner by GW501516 treatment (**Figure 7B**). Moreover, we also confirmed that the AMPK inhibitor compound C could abrogate the suppressing effect of GW501516 on ILK gene and protein expression (**Figures 7C,D**). These results suggests that GW501516 inhibited NPC cell growth and suppressed tumor formation in the BALB/c nu/nu mice may be associated with the activation of AMPK and the inhibition on ILK expression.

## Discussion

The current study disclosed a novel role for activation of PPARβ/δ by GW501516 in suppressing the proliferation and tumorigenicity of NPC for the first time. We found PPARβ/δ expression is reduced in the poor- and un-differentiated NPC cell lines. Which may be associated with the aggressive clinical behavior in NPC cells. Based on this, we further demonstrated PPARβ/δ activation can suppress the proliferation of NPC cells at both *in vitro* and *in vivo* level, which is realized by impairing cell cycle progression and promoting apoptosis through up-regulating apoptotic associated proteins caspase and BAX. The underlying molecular mechanism is related to GW501516’s inhibition on the expression of ILK after activation of the AMPKα. Results from this study thus provided new insights and evidences that PPARβ/δ plays crucial roles in inhibiting the tumorigenicity and progression of human NPC, and holds the potential as a molecular target for NPC intervention.

The expression level of PPARβ/δ in the NPC cells seems depend on the degree of differentiation, as revealed by QPCR and western blot in the four lines of commonly studied human NPC cell lines with different degree of differentiation (Chen et al., 2015). Among them, HK1 and CNE1 are confirmed to be well-differentiated (Huang et al., 1980), and CNE2 is poorly differentiated (Sizhong et al., 1983), whereas C666-1 is an undifferentiated and also an EBV positive NPC cell line (Cheung et al., 1999). PPARβ/δ mRNA level in the poor-differentiated CNE2 cells and the undifferentiated C666-1 cells were lowered to 76.6 and 53.7%, respectively, as compared with the control NP-69 cells. Correspondingly, protein expression of PPARβ/δ was strikingly reduced to 65.7 and 34.7% in these two cell lines. Whereas gene and protein expression of PPARβ/δ in the well differentiated EBV-negative NPC cells wasn’t abated. These results together suggested PPARβ/δ expression within the NPC cells seems in reverse correlation with the degree of differentiation of the NPC cells, to some extent, and lower PPARβ/δ expression was linked to a more aggressive phenotype of NPC. This finding is in agreement with the report that PPARβ/δ’s expression was lower in rectal cancers with poor differentiation than in those with better differentiation, and further corroborated previous reports that PPARβ/δ is responsible for the terminal differentiation of multiple type of cells by modulating expression of cell type specific differentiation-related proteins (Tan et al., 2001; Westergaard et al., 2001; Kim et al., 2006; Yang et al., 2010). Through induction of terminal differentiation, PPARβ/δ may suppress sustained cell growth and inhibit hallmark of cancer checkpoints (Peters et al., 2015a). Additionally, in consistent with current finding in the NPC, decreased expression of PPARβ/δ protein also had been found in numerous other types of tumors including colorectal, gliomas, melanoma, skin, urothelial cancers (Peters et al., 2015b).

The role of PPARβ/δ in cancer biology appears to be context dependent. Recently, numerous studies described increased or decreased expression of PPARβ/δ in multiple types of cancers, and also disclosed distinct roles for PPARβ/δ in tumor progression and invasion (Peters et al., 2015a). In current study, we analyzed the impact of PPARβ/δ activation on the growth of the NPC cells with a PPARβ/δ agonist, GW501516, who had shown anti-tumor effect on breast and skin tumors (Yao et al., 2014). As expected, the MTT assay disclosed different response of PPARβ/δ activation on the growth of NPC cells with different degree differentiation, and this response is related to the PPARβ/δ expression level within the cell. The most severe inhibition on cell proliferation was observed in the undifferentiated EBV-positive C666-1 cells, who had the lowest PPARβ/δ expression among the NPC cells assayed, and followed by the poor-differentiated CNE2 cells. We thus take the C666-1 cells in our further studies, which also had been widely regarded as the best cell model for recapitulating the biological behavior of human NPC (Xiao et al., 2007; Zhang et al., 2016). In line with the MTT assay, colony formation assay further demonstrated that GW501516 markedly inhibited colony formation in C666-1 cells. Additionally, the growth suppression effects induced by GW501516 was reverted by a PPARβ/δ antagonist GSK3787 (Shearer et al., 2010), further confirming the effects of GW501516 was realized through activation of PPARβ/δ. More importantly, specifically overexpression of PPARβ/δ in C666-1 cells also could mimic the beneficial effects of PPARβ/δ activation on the growth inhibition, further disclosed the specific role of PPARβ/δ in this process.

Through flow cytometry analysis, we further demonstrated that GW501516 impaired cell cycle progression, led to a G2/M phase arrest, and induced apoptosis in C666-1 cells. The results suggested that the observed growth-inhibition effect of PPARβ/δ activation or over-expression on NPC cells might be mediated through modulation of cell cycle progression. This is similar to the recent report in mouse keratinocytes expressing an oncogenic form of harvey sarcoma ras, where ligand activation of PPARβ/δ also led to a G2/M arrest of the cell cycle (Zhu et al., 2012). Meanwhile, this growth suppressing effect of PPARβ/δ in NPC cells is also similar to the previous reports on endometrial, urothelial and mouse mammary gland cancer cells (Péchery et al., 2016). Furthermore, *in vivo* studies in the BALB/c nu/nu mice xenograft model clearly demonstrated that GW501516 administration could suppress the growth of NPC significantly. Thus, the *in vitro* and *in vivo* results together suggested that GW501516 is effective in preventing the development and progression of undifferentiated NPC.

Apoptosis resistance is important for cell proliferation and tumor growth, and also an accepted hallmark of cancer. The apoptosis signaling pathway is mediated by caspases, and among them, caspase-3 and caspase-9 are crucial mediators in the apoptosis procedure. The apoptosis pathway can be initiated through caspase-9, and trigger apoptosis through the cleavage of the downstream executioner caspase-3, and thereafter the direct executant PARP (McDonald et al., 2008; Zheng et al., 2009). Bcl-2 and Bax are two typical proteins of the Bcl-2 family that play critical roles in caspase-dependent apoptosis. The former is an anti-apoptotic protein, and the latter is an apoptosis promoting protein. Bcl-2 could inhibit the Bax-induced caspase-dependent apoptosis, and thus the ratio of Bcl-2/Bax within cells ultimately determines the cell fate. Here in the GW501516 treated C666-1 cells, we found that the level of Bcl-2 was decreased, while the expression of Bax, caspase-3, and caspase-9 were increased strikingly, and together with the significantly enhanced ratio of Bax to Bcl-2. However, caspase-8 expression was not altered under such condition, indicating the death receptor mediated apoptotic pathway was not included in this process (Park et al., 2003). More importantly, elevated expression of caspase-3 and caspase-9, and the ratio of Bax to Bcl-2 were further confirmed in the GW501516 treated C666-1 xenograft samples. Furthermore, in the same sample, we also find exorbitantly up-regulated phosphorylation of AMPKα and down-regulated ILK by GW501516. This is similar to the regulation of PPARγ agonist rosiglitazone and metformin on suppressing ILK gene expression in inhibition the growth of CNE1 NPC cells by activation of AMPKα signaling pathway (Hahn et al., 2014). ILK is a well-recognized cancer cell survival promoting protein, and its overexpression is associated with enhanced tumorigenicity (McDonald et al., 2008; Zheng et al., 2009). In contrast, ILK inhibitor could attenuate growth of head and neck cancer cells (Younes et al., 2007; Eke et al., 2009). Thus it can be concluded that the suppression of ILK after AMPKα activation caused by GW501516 is connected to induce NPC cell apoptosis and growth inhibition. However, the underlying relations among them needs further study to clarify.

## Conclusion

The results of the present study on NPC cells and xenograft mice indicated that PPARβ/δ activation by GW501516 exerts direct antiproliferative effect via induction of G2/M phase arrest and apoptosis promoting effect by modulating the expression of the mitochondria controlled apoptotic pathway (**Supplementary Figure S1**). Which is greatly associated with its inhibition on the expression of ILK after activation of the AMPKα-dependent signaling pathways. Thus PPARβ/δ activation may provide a novel therapeutic strategy for the treatment of NPC, particularly to those histopathological classified as poor to undifferentiated. However, the detailed mechanism and pathways by which PPARβ/δ regulate the cell cycle and apoptosis still needs further study. We will further address these unraveled questions and explore if current results could be recapitulated in clinical NPC tissues in the following work.

## Author Contributions

YJ and LG conceived and designed the experiments and wrote the paper. YJ, HL, and FW performed the experiments. YJ, HL, FW, and LG analyzed the data.

## Conflict of Interest Statement

The authors declare that the research was conducted in the absence of any commercial or financial relationships that could be construed as a potential conflict of interest.

## Supplementary Material

FIGURE S1 The antitumor mechanistic scheme of PPARβ/δ agonist GW501516 in C666-1, an un-differentiated nasopharyngeal carcinoma cell line. Dotted line indicated further work is needed to exactly elucidate the connection.
