# Supplementary figures and images for: PPARβ/δ Agonist GW501516 Inhibits Tumorigenicity of Undifferentiated Nasopharyngeal Carcinoma in C666-1 Cells by Promoting Apoptosis
Source: Front Pharmacol. 2018 Jun 28;9:648. doi: 10.3389/fphar.2018.00648 (PMC6031703; doi:10.3389/fphar.2018.00648)

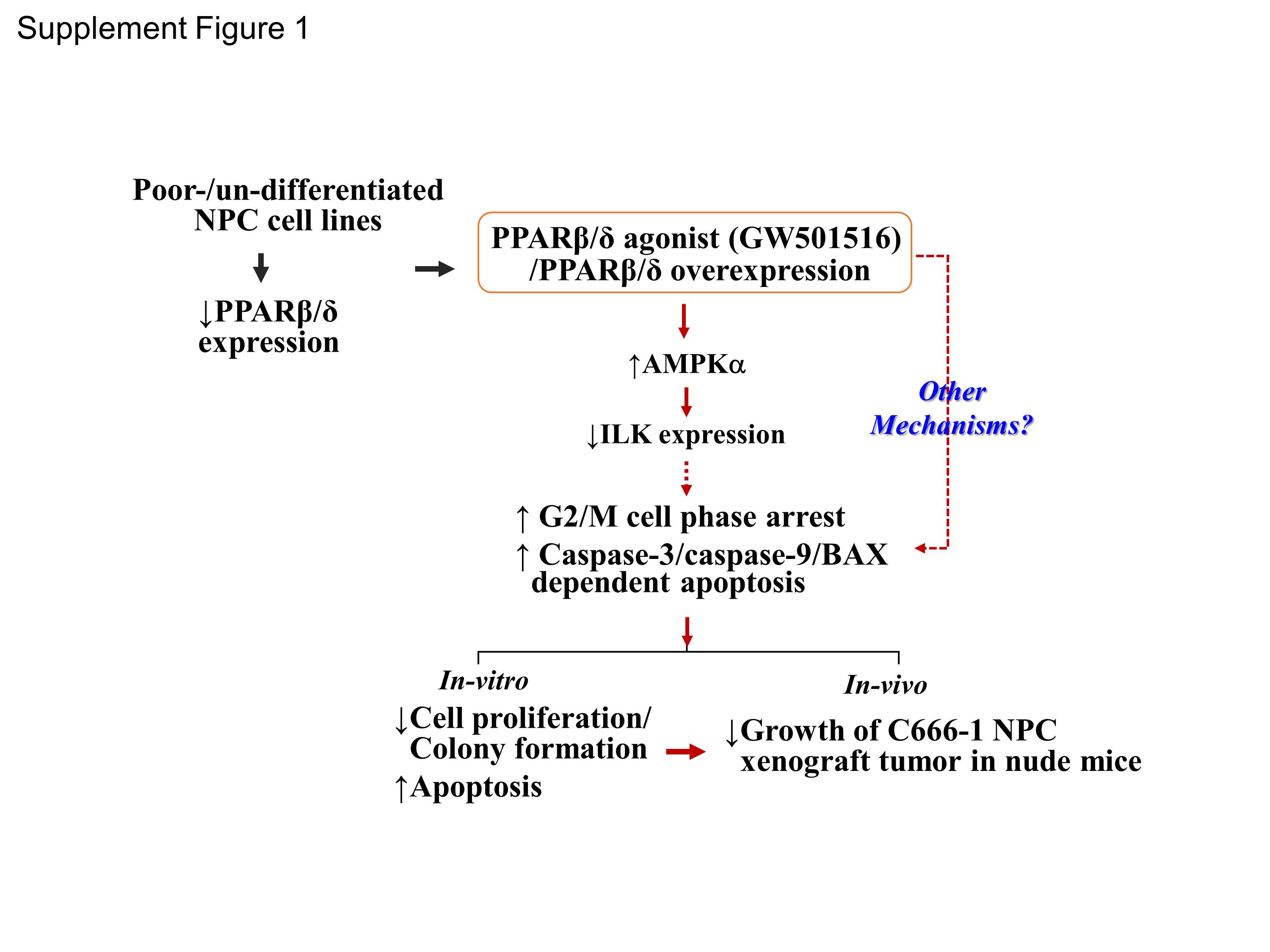

Supplement: FIGURE S1 — The antitumor mechanistic scheme of PPARβ/δ agonist GW501516 in C666-1, an un-differentiated nasopharyngeal carcinoma cell line. Dotted line indicated further work is needed to exactly elucidate the connection. [file Image_1.JPEG]
